# Anti-Phosphatidylserine, Anti-Prothrombin, and Anti-Annexin V Autoantibodies in Antiphospholipid Syndrome: A Real-Life Study

**DOI:** 10.3390/diagnostics13152507

**Published:** 2023-07-27

**Authors:** Daniele Roselli, Maria Addolorata Bonifacio, Giovanna Barbuti, Maria Rosaria Rossiello, Prudenza Ranieri, Maria Addolorata Mariggiò

**Affiliations:** 1 Department of Precision and Regenerative Medicine and Ionian Area, University of Bari Aldo Moro Medical School, 70124 Bari, Italy; rosellidaniele94@gmail.com (D.R.); m.bonifacio@studenti.uniba.it (M.A.B.); giovanna.barbuti@uniba.it (G.B.); mariarosaria.rossiello@uniba.it (M.R.R.); 2 Department of Precision and Regenerative Medicine and Ionian Area, Section of Experimental and Clinical Pathology, Azienda Ospedaliero-Universitaria Consorziale Policlinico di Bari, 70124 Bari, Italy

**Keywords:** antiphospholipid syndrome, phosphatidylserine, prothrombin, annexin-V, antiphospholipid antibodies, lupus anticoagulant, autoimmune disease

## Abstract

The antiphospholipid antibodies (aPL) increase the risk of developing thrombotic events and may coexist with a variety of autoimmune diseases. They can be detected chronically or temporarily in patients with infectious diseases, during drug therapy, or in cases of cancer. A thrombotic event with aPL detection is known as antiphospholipid syndrome (APS) and the diagnostic criteria include the presence of lupus anticoagulant (LA), anticardiolipin (aCL) and β_2_-glycoprotein-1(aβ_2_GPI) antibodies. Other autoantigens recognized in APS are phosphatidylserine (aPS), prothrombin (aPT) and Annexin-5 (aA5). This real life study aimed to explore the connections between laboratory criteria and the prevalence of “non-criteria aPL” in APS. This study followed 300 patients with thrombosis and employed two phospholipid sensitivity assays for LA detection, chemiluminescence assays for aCL and aβ_2_GPI and enzyme-linked immunoassays for aPS, aPT and aA5. A significant association was found between aPS and aCL (r = 0.76) as well as aβ_2_GPI (r = 0.77), while the association with LA was less significant (r = 0.33). The results of the aPT and aA5 test did not correlate with criteria-antiphospholipid antibodies (r < 0.30). Since the risk of thrombotic complications increases with the intensity and the number of positive autoantibodies, measuring aPT and aA5 autoantibodies may be useful, particularly in aCL/aβ_2_GPI-negative patients or in cases of isolated LA positivity.

## 1. Introduction

Antiphospholipid syndrome (APS) is a systemic autoimmune disease characterized by thrombosis and pregnancy morbidity in the presence of persistently positive antiphospholipid (aPL) antibodies [1]. APS can cause neurological disorders (e.g., impaired cognitive function or demyelination signs) not directly related to thrombotic lesions [2], although the association with multiple sclerosis, epilepsy [3], or neuropsychiatric lupus [4] is defined as secondary antiphospholipid syndrome. The relationships between APS and unexplained thrombocytopenia, haemolytic anaemia, livedo reticularis, or other autoimmune diseases have been investigated [5]. These autoantibodies are usually directed against specific proteins that are able to bind phospholipids (e.g., β_2_-glycoprotein-1, prothrombin and annexin-5) expressed by various cell types (e.g., endothelial cells, monocytes and platelets). The production of autoantibodies is an abnormal response of the immune system against itself, during which the immune system elicits the clonal expansion of B lymphocytes as a reaction to epitopes present in infectious agents [6]. The secreted autoantibodies might attack tissues, including vital organs. Viruses [7] and other microbial agents may induce an autoimmune disease through several mechanisms, triggering the production of aPL through molecular mimicry [8].

The aPL antibodies promote platelets activation mediated-complement deposition [9], induce a proinflammatory and procoagulant endothelial phenotype, upregulating cellular adhesion molecules and supporting the synthesis of proinflammatory cytokines such as interleukin-1 and TNF-α [10].

Within the clotting cascade, the phosphatidylserine has a specific interaction with the membrane binding γ-carboxyglutamic acid residues (Gla-domain) of protein substrates (i.e., FVII, FX). These interactions with the membrane surface explain how PS molecules can provide the basis for strong and specific binding between a membrane surface and a water-soluble protein. The Gla-dependent binding of both enzyme and substrate to a PS-containing membrane increases catalytic efficiency by three orders of magnitude [11]. Therefore, the exposure of PS on the surface of apoptotic cells is important not only because it is the basis of an effective clearance process, but also because that process is immunologically silent. Disorders in the elimination of apoptotic cells are associated with autoimmune diseases, i.e., systemic lupus erythematosus and rheumatoid arthritis [12,13]. These events may support a variety of other conditions, including neurological diseases and gynaecological issues [14]. In an effort to expand the panel of aPL for APS diagnostics, we studied antibodies against phosphatidylserine (aPS). These antibodies inhibit the development and invasion of the trophoblast, decrease hCG levels, and delay the formation of syncytiotrophoblast in in vitro models [15].

The annexin-5 belongs to the family of Ca^2+^-dependent proteins binding phospholipids and it is the main component of trophoblast and vascular endothelia [16]. Annexin-5 binds phospholipids with anticoagulant activity and acts as a protective shield to conceal exposed phospholipid surfaces. This shield may be impaired during the interaction of A5 with antibodies, leading to thrombosis and miscarriages [17]. Moreover, A5 has proved very useful as a marker for apoptosis and platelets activation [18]. During the apoptotic process, particularly when PS is exposed on the cell surface, A5 binds to the apoptotic cells, inhibiting their procoagulant and pro-inflammatory activities.

The prothrombin is activated by the tenase complex (i.e., factor Xa, factor V, calcium and phospholipids) during the penultimate step of the coagulation process, i.e., the stabilisation of platelet aggregates through the cascade activation of proenzymes belonging to the intrinsic and extrinsic pathways [19]. The transformation of prothrombin into thrombin occurs on activated cell membranes such as platelets. Anti-prothrombin antibodies may reduce the level of prothrombin and determine bleeding diathesis [20]. It is possible to observe a prolonged prothrombin time and an activated partial thromboplastin time, which are not corrected in mixing studies with normal plasma. This is due to the presence of aPL antibodies in patients with thrombotic symptoms rather than bleeding symptoms. The incomplete and heterogeneous diagnostic tests currently available make the diagnosis of antiphospholipid syndrome challenging.

The international consensus for APS classification includes the lupus anticoagulant test (LA) in the laboratory criteria, showing the highest strength of association with thrombotic complications. Furthermore, the consensus criteria also involve the detection of anti-cardiolipin (aCL IgG/IgM) and anti-β_2_-glycoprotein I (aβ_2_GPI IgG/IgM) antibodies. Sometimes, the LA functional assay does not associate with aPL and its positivity remains isolated [21] or the thrombosis has not yet manifested. The “seronegative APS” was recognized as a distinctive setting [22] or was better redefined by the demonstration of new classes of antiphospholipid antibodies directed toward clotting proteins (i.e., prothrombin and annexin-5, acting as phospholipids cofactors). An increased understanding of aPL for specific immune targets along with LA provide an added value to the classification criteria.

Since it is very hard to identify the clinical significance of low titres of aPL, of single positivity, or of weak LA activity, it would be useful to test other autoantibodies in APS profiling.

We herein report the results obtained after the introduction of aPS, aPT, and aA5 antibodies in our clinical laboratory practice. We analysed the prevalence of these antibodies, their relationship with aCL and aβ_2_GPI antibodies, their association with LA, and their additional contribution to the APS diagnostic process.

## 2. Materials and Methods

### 2.1. Patients Characteristics

In this study, 300 patients with thrombotic manifestations presented a medical prescription for aPL antibodies. The cohort included 107 males (36%) and 193 females (64%), with a median age of 53 years. The patients, recruited from a real-life clinical practice, were not on oral anticoagulant therapy, did not have hereditary thrombophilia, and underwent functional testing.

### 2.2. Laboratory Methods

Initial testing is performed shortly after a clinical event, when it may influence the treatment decision. However, it is preferably performed away from the acute phase of venous thromboembolism [23]. In order to provide background information, i.e., unexpected coagulopathies or undocumented anticoagulation therapies, routine clotting tests were performed on citrated plasma. The prothrombin time (PT, Dade^®^ Innovin, Siemens; reagent prepared from purified recombinant human tissue factor), the activated partial thromboplastin time (aPTT, Dade^®^ Actin FSL Activated, ellagic acid activator reagent; Siemens, Bari, Italy), and fibrinogen determination (Clauss method, Dade^®^ Thrombin Reagent, Siemens, Bari, Italy) were all carried out on a Sysmex CS 5100 coagulation analyser (Siemens, Bari, Italy). The presence of LA was tested through a three-reflex procedure including screening, mixing, and confirmation assays. The Silica Clotting Time Test (SCT, Hemosil Silica Clotting Time, Instrumentation Laboratory, Milan, Italy) and the dilute Russel’s Viper Venom Test (dRVVT, Hemosil dRVVT, Instrumentation Laboratory, Milan, Italy) were performed on the ACL TOP^®^ System (Instrumentation Laboratory, Milan, Italy) at two different concentrations of phospholipids. LA negative control plasma (PNP, Hemosil Instrumentation Laboratory, Milan, Italy) was used in the mixing test (patient plasma/PNP, at 1:1 ratio). The LA test was considered positive if either the SCT or dRVVT assays yielded a ratio higher than 1.20 in the confirmatory test. Similar to Devreese [24], LA results were graded as follows, i.e., 0 (both assays negative), 1 (one positive assay), 2 (two positive assays) and 3 (two positive assays with prolonged aPTT and/or PT).

The detection of aCL and aβ_2_GPI autoantibodies, belonging to the IgG/IgM isotype, was performed with a chemiluminescence immunoassay method (Zenit RA Menarini Diagnostics kit). We set medium to high titre values (>40 GPL or MPL, or >99th percentile) to determine aCL and aβ_2_GPI positivity. For low positivity, the cut-off value ranged from 10 to 40 GPL or MPL. Serum aPT, aPS, and aA5 autoantibodies were measured using an enzyme-linked immunoassay (Orgentec Diagnostika GmbH, Mainz, Germany). Briefly, highly purified prothrombin or human annexin-5 were bound to microwells for the detection of aPT and aA5 antibodies, respectively. Purified phosphatidylserine was saturated with β_2_glycoprotein I. The results for aPS, aPT, and aA5 were obtained by reading the measured optical density from the calibration curve. The cut-off level for positivity was set at >10 U/mL for IgG and IgM antibodies.

### 2.3. Statistical Analyses

Statistical analyses were performed using R software, version 4.0.1. Relationships between the levels of aPS, aPT, aA5, aCL, and aβ_2_GPI antibodies and LA, graded from 0 to 3, were compared using the Spearman’s correlation test. *p* values less than 0.05 were considered statistically significant.

## 3. Results

Among the 300 patients with thrombosis, 65 were seronegative for aPL antibodies, non-criteria antibodies, and LA. The number of positive samples was 235 (80%), with 81 (34%) having a prolonged aPTT and 12 (5%) showing both a prolonged PT and aPTT. As shown in Table 1, according to the laboratory classification criteria for APS [25], patients were divided into three different profiles: triple-positive (strong LA test, aCL and aβ_2_GPI antibodies exceeding the 99th percentile), double-positive (aCL and aβ_2_GPI titres exceeding the 99th percentile and LA negative), single-positive (isolated LA, abnormal SCT and/or dRVVT, aCL/aβ_2_GPI below the 99th percentile). A triple positivity profile for aPL was detected in 45 patients (19%), double positivity in 15 (6%), and single positivity in 175 (74%). The distribution of autoantibodies and LA is shown in Table 1. In this regard, aCL were the most frequently detected antibodies in the three patient groups, followed by aβ_2_GPI antibodies. Thus, the observed cohort confirmed the high occurrence of the criteria antibodies, as expected. Nevertheless, aPS antibodies were also frequently detected, especially in triple-positive patients.

This outcome was confirmed on the whole cohort through the study of the correlation between non-criteria aPL (aPS, aPT, aA5 IgG/IgM), aPL criteria (aCL, aβ_2_GPI), and LA, as reported in Table 2. Indeed, aPS antibodies showed a Spearman’s correlation coefficient higher than 0.75 with aCL and aβ_2_GPI antibodies. On the other hand, the correlation between aPS and LA assay was weak (r = 0.21). Similar results were obtained for aPT in correlation with criteria antibodies and LA assay (r = 0.20, 0.21 and 0.21, respectively). Concerning aA5 antibodies, no correlation emerged from the performed assessments.

**Figure 1:**
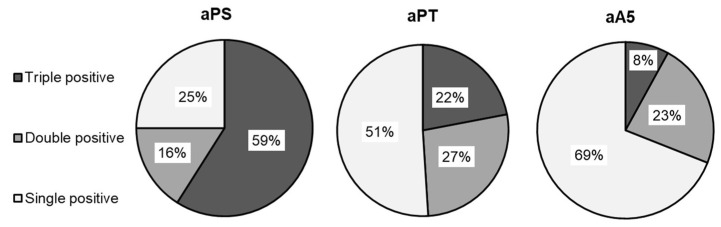
Prevalence of non-criteria antibodies (aPS, aPT and aA5) in triple, double, and single-positive patients.

Therefore, we focused on the non-criteria antibodies, observing their prevalence in more detail. Figure 1 shows the prevalence rates of aPS, aPT, and aA5 antibodies within the different groups of patients. More than 50% of aPS antibodies were detected in the triple positive cohort. Additionally, in single positive patients, the aPS antibodies were associated with low levels of aCL/aβ_2_GPI (35 and 37%, respectively). Furthermore, it is worth noting that, within single-positive patients, aPS antibodies often occurred with high levels of aPT antibodies (52%). The latter appear to be independently occurring antibodies in the studied cohort, not associated with the standard aPL profile (aCL and aβ_2_GPI correlation coefficient r~0.20, Table 2). Similarly, the aA5 antibodies showed high prevalence (69%) in single positive patients. Despite the limitations of a small patient cohort, these findings are in agreement with the literature, highlighting the potential usefulness of aPT and aA5 antibodies in symptomatic patients displaying few diagnostic laboratory criteria [26,27].

## 4. Discussion

The diagnosis of antiphospholipid syndrome is part of a routine clinical practice to detect acquired risk factors for thrombotic events. Literature data report a clinical heterogeneity of the APS and aPL laboratory phenotype, although the thrombotic risk is increased in patients with strong LA, high aCL, and aβ_2_GPI levels [28].

Interestingly, in our cohort, only 19% of the results showed triple positivity (Table 1), with prevalence rates for aPS/aPT/aA5 antibodies of 59, 22, and 8%, respectively (Figure 1). Moreover, our data (Table 2) show a significant correlation of aPS IgG/M antibodies with aCL, and aβ_2_GPI (r = 0.76 and 0.77, respectively). There are no patients with isolated aPS positivity, although aPS antibodies are more frequently found in triple and double positive patients. In this respect, Park et al. utilized line immunoassay detection to demonstrate that single positivity of aPS is a better predictive factor for thrombosis than aβ_2_GPI positivity [29]. Monoclonal antibodies against phosphatidylserine inhibit in vitro human trophoblastic hormone production and invasion [15] but their utility is controversial, due to cross reaction with other aCL antibodies [30]. Based on previous literature findings, as well as on the outcomes of our study, we hypothesize that the presence of aPS may be redundant for diagnosing APS, especially in triple positive patients, in which its correlation with aCL and aβ_2_GPI is very high.

Possible target proteins for aPL include prothrombin and annexin-5. The detection of aPT is associated with pregnancy loss in females with primary APS [31]. Additionally, Palosuo et al. reported that aPT constitutes an independent risk factor for venous thromboembolism [32]. Moreover, high levels of aPT predict a 2.5 times increased risk of cardiovascular events [33] and have been associated with disease severity in patients with SARS-CoV-2 [34]. Forastiero et al. found a higher rate of thrombosis in patients with positive aPT when compared with patients without aPT [35]. Furthermore, the results on a 15-year longitudinal study [36] confirmed that aPT antibodies and LA positive results are the most useful predictors of thrombosis in systemic lupus erythematosus (SLE). Since it has been reported that aPS/aPT are useful diagnostic markers of thrombosis in APS [37], our study highlights that the aPT antibodies appear to exhibit independent positive behaviour. This means that there is no correlation between aPT and aCL/aβ_2_GPI antibodies (r = 0.20 and 0.21, respectively). In single positive samples, the prevalence rates for aPS/aPT/aA5 antibodies are 26, 52, and 69% respectively (Figure 1). Of single positive APS patients, 21% show anti-prothrombin or aA5 antibodies associated with strong LA. Interestingly, seven patients with abnormal PT tests were positive for aPT antibodies, even though they had no clinical bleeding. Thus, it is likely that the detected IgGs could interfere with different prothrombin epitopes. The diagnostic immunoassay herein performed (ELISA Orgentec Diagnostika GmbH, Mainz, Germany) does not allow the determination of the epitopes targeted by patients’ aPT antibodies. Indeed, as reported by Atsumi and coworkers, aPT could target cryptic epitopes, as well as neoepitopes exposed after prothrombin binding to other phospholipids [38]. In this regard, Chinnaraj and coworkers recently proposed a novel ELISA assay to help identify the epitopes targeted by aPT antibodies [39]. They synthesized a biotinylated prothrombin variant, immobilised on neutravidin-coated plates in a precise density and orientation, eliminating the need for phospholipid-based reagents that often cause standardization issues. The authors successfully correlated the outcomes of their developed ELISA assay with those of the INNOVA kit for aPS/PT detection, testing 27 triple-positive APS patients. The cost-effective and reproducible technique developed by Chinnaraj et al., once validated on larger patient cohorts, could be applied to develop more specific ELISA assays, shedding light on the epitopes recognized by APS-relevant autoantibodies. A more detailed research study, exceeding the scope of this real-life clinical survey, could provide clarification regarding the target epitopes of aPT, aPS, and aA5 antibodies. However, in APS patients who are positive for aPT and undergoing oral anticoagulant therapy, follow-up control with PT-INR might be challenging. When LA, in addition to aβ_2_GPI or aPT antibodies, is detected, the risk of thrombosis increases by a further three times [37]. Therefore, aPT antibodies could be an added value for accurate APS diagnosis in these patients, and their presence may change the classification criteria from single to strong positivity.

Moreover, in this study, only 13 patients were positive for aA5, who were mainly male, and the highest prevalence was observed in the single positive samples (69%), which did not always overlap with LA or other markers. It has been reported that annexin-5 forms a protective anticoagulant shield on vascular endothelial cells and that aβ_2_GPI antibodies may disrupt the shield, predisposing to thrombosis [40]. Considering the high variability of the reported results, it remains controversial whether aA5 antibodies are associated with thrombotic manifestations or impaired immune system conditions. Indeed, Singh et al. found aA5 positivity in 69 out of 112 APS patients and only 3 out of 40 healthy controls [41]. On the contrary, de Laat et al. found no association between aA5 and history of thrombosis in 198 patients with primary APS, SLE, or lupus-like disease [42]. Furthermore, aA5 antibodies seem to have a role in COVID-19-associated APS syndrome [43]. Our data show that annexin-5 seems to be an independent antibody, not correlated with aPL criteria (r = 0.00). However, due to their low presence, a larger prospective cohort study in a selected, homogeneous population is needed to clarify the role of aA5 in patients with thrombosis.

Furthermore, in previously proposed criteria for APS [23], the presence of an aPS/aPT antibodies complex characterizes the tetra-positivity profile. In this study, the data from the three patient groups support these proposed criteria. Specifically, in triple positivity, 24% of patients are also positive for aPT. Among those with double-positivity, the aPT/aA5 antibodies are associated with aPL-criteria in 21% of cases. Finally, in the single-positivity group, aPT occurs in conjunction with LA in 8% and with aPL in 13% of cases.

Therefore, in the case of suspected LA outcomes, other tests should be conducted (e.g., aPT or aA5 assay). In this scenario, the LA assay could be considered a comprehensive test for aPL and should always be correlated with the aPL results, as well as with the patient’s clinical history and the timing of the assay, in order to assess the risk profile.

Moreover, our data provide further evidence that aPTT with an ellagic-acid activator reagent is prolonged only in patients with higher titres of antibodies (28%), and a weak LA may not be considered predictive of APS. Indeed, the SCT/dRVVT screening and confirmatory tests are sensitive and specific for LA, although a false-positive LA diagnosis is not uncommon [44]. Thus, it is not excluded that transiently elevated levels of antibodies can occur in the setting of some infections or drug exposures. Additionally, further research should be dedicated to correlating each specific autoantibody panel with the corresponding severity of thrombotic events.

Taking our results together, it can be concluded that antiphospholipid syndrome remains a major challenge for clinicians and pathologists, who struggle to prevent misclassification and provide appropriate therapies. Therefore, patients with low titres of aCL/aβ_2_GPI or weak LA should be closely monitored, considering the seriousness of the outcomes. It is important to emphasize that these additional antibodies could be included in routine laboratory tests to recognize unconventional APS.

## 5. Conclusions

We hypothesized that aPS assay may be redundant due to its high correlation with aCL and aβ_2_GPI. However, a complete antibody profile holds significant prognostic importance regarding the risk of thrombosis. Non-conventional aPL, such as aPT and aA5, could demonstrate high diagnostic applicability and may be relevant for thromboprophylaxis, as well as understanding new physiopathogenic mechanisms in unconventional APS.

## Figures and Tables

**Table 1:** Distribution of autoantibodies IgG/IgM and LA positivity in three different cohorts of APS patients.

	Triple-Positive *n* = 45 (19%)	Double-Positive *n* = 15 (6%)	Single-Positive *n* = 175 (74%)
aCL IgG, *n* (%)	40 (89)	10 (67)	5 (3)
aCL IgM, *n* (%)	5 (11)	4 (27)	33 (19)
aβ_2_GPI IgG, *n* (%)	38 (84)	10 (67)	8 (5)
aβ_2_GPI IgM *n* (%)	7 (15)	3 (20)	30 (17)
aPS IgG, *n* (%)	37 (82)	8 (53)	4 (2)
aPS IgM, *n* (%)	5 (11)	4 (27)	17 (10)
aPT IgG, *n* (%)	10 (22)	2 (13)	20 (11)
aPT IgM, *n* (%)	3 (6)	1 (7)	9 (5)
aA5 IgG, *n* (%)	1 (2)	2 (13)	3 (2)
aA5 IgM, *n* (%)	0	1 (7)	5 (3)
LA, *n* (%)	45 (100)	0	154 (88)

**Table 2:** Correlation titres of aPL non-criteria (aPS, aPT, aA5 IgG/IgM), aPL criteria (aCL, aß2GPI), and LA assay grades. Spearman’s correlation coefficient (r).

	r
aPS/aCL	0.76
aPS/aβ_2_GPI	0.77
aPS/LA	0.21
aPT/aCL	0.20
aPT/aβ_2_GPI	0.21
aPT/LA	0.21
aA5/aCL	0.00
aA5/aβ_2_GPI	0.00
aA5/LA	0.00

## Data Availability

All reported data are herein available. Raw data are available from the corresponding authors, on reasonable request.
